# Early versus delayed fixation of maxillofacial fractures in polytrauma: a systematic review

**DOI:** 10.1007/s00068-026-03127-2

**Published:** 2026-02-23

**Authors:** Jane Chen, Karen Vuong, Zsolt J. Balogh

**Affiliations:** 1 Department of Oral Maxillofacial Surgery, Monash Health, Melbourne, VIC Australia; 2 Department of Plastic and Reconstructive Surgery, Western Health, Melbourne, VIC Australia; 3 Department of Traumatology, John Hunter Hospital, Newcastle, NSW Australia; 4 Discipline of Surgery, School of Medicine and Public Health, University of Newcastle, Newcastle, NSW Australia

**Keywords:** Polytrauma, Multiple injuries, Maxillo-facial trauma, Trauma centre, Trauma system, Trauma surgery

## Abstract

**Background:**

Maxillofacial fractures are common in polytrauma, and these patients often require intensive care unit (ICU) admission until delayed definitive surgical management. This review aims to evaluate the scientific evidence on timing of definitive internal fracture fixation of maxillofacial fractures in polytrauma. We hypothesise that delayed definitive surgery is associated with better outcomes.

**Methods:**

This systematic review was conducted in accordance with the PRISMA protocol. MEDLINE, EMBASE, Scopus, CINAHL and PubMed were searched from inception to July 2025 for articles in English reporting timing of facial fracture fixation in polytrauma cohorts. Two reviewers independently screened studies and extracted data on patient characteristics, injury severity, fixation timing, and patient outcomes. Risk of bias was assessed using the Newcastle-Ottawa Scale.

**Results:**

359 studies were initially identified. After applying inclusion and exclusion criteria, three retrospective studies (total 365 patients) were included. Timing definitions of fixation varied, with early fixation intervals ranging from ≤ 24 h to ≤ 72 h. Surgical-site infection rates ranged from 0 to 12.7% (early) and 4.8–14.4% (delayed). No significant differences emerged in anatomical reduction, reoperation rates, or complications between groups. Hospital and ICU length of stay, ventilator days and mortality rates were comparable. Studies were heterogeneous and subject to high risk of bias.

**Conclusions:**

The available low-quality evidence does not support a clear advantage of the current practice of delayed fixation of facial fractures in polytrauma patients. Quality prospective studies with predefined inclusion criteria, clear consensus about the cut-off for early timing of surgery and standardised outcome measures are required.

**Supplementary Information:**

The online version contains supplementary material available at 10.1007/s00068-026-03127-2.

## Background

Polytrauma is a disease which is defined by severe injuries to multiple body regions, physiological compromise, systemic inflammatory response, high risk for uninjured organs to fail and disproportionally poor outcomes compared to the expected outcomes based on injuries only [1–3]. Maxillofacial trauma occurs frequently as part of polytrauma, with a reported prevalence of 18.5–50% [4–7]. Injuries to these structures may result in life-threatening haemorrhage [8, 9] and disturb critical physiological functions such as mastication, speech, ocular movements, brain function and airway patency [10, 11], which necessitates the need for ICU admission, airway protection with intubation [6] or tracheostomy [11]. Patients are therefore predisposed to septic and respiratory complications, such as sinus infection and ventilator associated pneumonia [7]. Furthermore, compromised facial aesthetics negatively affect psychosocial aspects of patients’ health, contributing to long term morbidity [12]. Surgical management should therefore aim to restore the form, function and aesthetics of the injured structures in a timely and anatomically precise manner.

The optimal timing of fixation of maxillofacial fractures has been disputed and literature is predominantly focussed on outcomes of isolated facial fractures [13]. Mandibular fractures are recommended to be repaired early as reasonably possible [14]. However, a lack of optimal operating room access within the context of resource-limited hospital systems resulting in the prioritisation of more time-dependent interventions and/or cost considerations [15], may result in the deferral of operative management. In certain circumstances fracture repair may even be delayed up to a week or are electively repaired following outpatient triage. There is currently a lack of standardisation within the literature as to what constituents as early versus delayed treatment, however, the evidence seems to suggest that a delay of up to several days to operative management is not associated with the development of significant complications in mandibular fractures [16–18]. Traditionally, for midface fractures without entrapment of the extraocular eye muscles or evidence of retrobulbar haematoma, definitive surgical fixation can be delayed until improvement of soft tissue oedema. In these circumstances, the injury to fixation timeframe is approximately 14 days [19–21]. However, early definitive fixation has been advocated by various authors, citing a reduced risk of surgical complications, such as surgical site infections [22, 23], technical difficulties and patient discomfort [13]. In the polytrauma population, timing the management of facial fractures is additionally complicated by the presence of severe injuries in other body regions, a widespread systemic inflammatory response, the potential organ dysfunctions or failures [24–29], prolonged local swelling as well as local and generalised septic complications.

Advances in trauma resuscitation have promoted the adoption of early, even pre-hospital haemostatic resuscitation strategies that prioritise balanced blood component administration and restrict large volume crystalloid infusions [30]. These strategies utilise blood product ratios approximating whole blood (1:1:1 of plasma to platelets to red blood cells) [31] to maintain haemostatic potential whilst limiting crystalloid use, thereby minimising post-injury complications secondary to tissue oedema, fluid overload and dilutional coagulopathy [32, 33]. An optimised physiological environment through improved resuscitation techniques provides an opportunity to reassess the benefits of early definitive surgical fixation compared to the historical strategy of delayed fixation, with the aim of reducing ICU stay, ventilator dependency, and other complications traditionally associated with delayed intervention [7, 28, 34–39].

Favourable outcomes for early fracture fixation in appropriately stabilised polytrauma patients with severe musculoskeletal injuries, such as spine, long bones and pelvic/acetabular fractures has been demonstrated, with reduced intensive care unit length of stays, and reduced pulmonary complication rates [28, 34–39]. Despite this, the applicability of improved patient outcomes through early surgical fixation in polytrauma patients or patients admitted to ICU with maxillofacial fractures has not been widely investigated. This systematic review aims to evaluate whether early definitive surgical fixation of maxillofacial facial fractures in polytrauma patients, compared to delayed surgical fixation improves clinical outcomes. We hypothesise that the current preferred practice of delayed definitive surgical care leads to better outcomes.

## Materials and methods

This systematic review was conducted in accordance with the Preferred Reporting Items for Systematic Review and Meta-analyses (PRISMA) 2020 guidelines [40]. A review protocol was registered with the PROSPERO database (CRD420251123521).

### Search strategy

A comprehensive search was conducted in the following electronic databases: MEDLINE, EMBASE, Scopus, Cumulative Index to Nursing and Allied Health Literature (CINAHL) and PubMed, and the searches were performed from database inception through to July 2025. The search strategy comprised of a combination the following terms which encompassed the area of interest (maxillofacial fractures, surgical fixation, timing and polytrauma): “maxillofacial injuries”, “facial fractures”, “zygomatic fracture”, “Le Fort fracture”, “nasal bone”, “orbital fracture”, “mandibular fracture”, “fracture fixation”, “surgical repair”, “open fracture reduction”, “internal fixation”, “early repair”, “early surgery”, “delayed repair”, “delayed surgery”, “treatment delay”, “treatment outcome”, “occlusion”, “anatomical reduction”, “postoperative complications”, “infection”, “malunion”, “function”, “diplopia”, “reoperation”, “multiple trauma”, “multitrauma”, “polytrauma”. A detailed table of the search strategy is attached as a supplementary table below. Additional relevant articles were identified by searching the reference list of selected articles.

### Eligibility criteria

Study types included were randomised control trials (RCT), prospective or retrospective cohort studies, case-control studies and large case series (*n* > 10) published in English with full text available, reporting on clinical outcomes (including anatomical reduction, functional outcomes, aesthetic outcomes, surgical complications and healthcare related outcomes) associated with different timing strategies for facial fracture fixation in a polytrauma patient were included. Articles reporting exclusively on frontal sinus, cranial base, temporal bone fractures or isolated dentoalveolar fractures were excluded. Nonclinical studies, case reports, conference abstracts, and technical notes were also excluded.

### Data selection & collection

Titles and abstracts were independently screened by two individuals. The full text of relevant articles was retrieved and further assessed by two independent reviewers against the inclusion and exclusion criteria. Data extracted included study characteristics (title, authors, year, geographic location, study design), patient demographics (age, sex, facial fracture type(s) and location, mechanism of injury, concurrent injuries and severity), treatment (time to treatment, operative management undertaken) and outcomes (functional, aesthetic, surgical outcomes, complications, length of stay, duration of follow-up and other associated outcomes). Conflicts were resolved via discussion between reviewers. Risk of bias was assessed via the Cochrane RoB-2 [41] for RCTs and given the expected paucity of RCTs, the Newcastle-Ottawa Scale [42] for observational studies.

### Data synthesis and analysis

A structured narrative synthesis of findings with descriptive statistics will be undertaken due to the small number of studies and anticipated heterogeneity in study designs, patient population, fracture subtypes, timing to treatment definitions and outcomes reported.

## Results

**Fig. 1:**
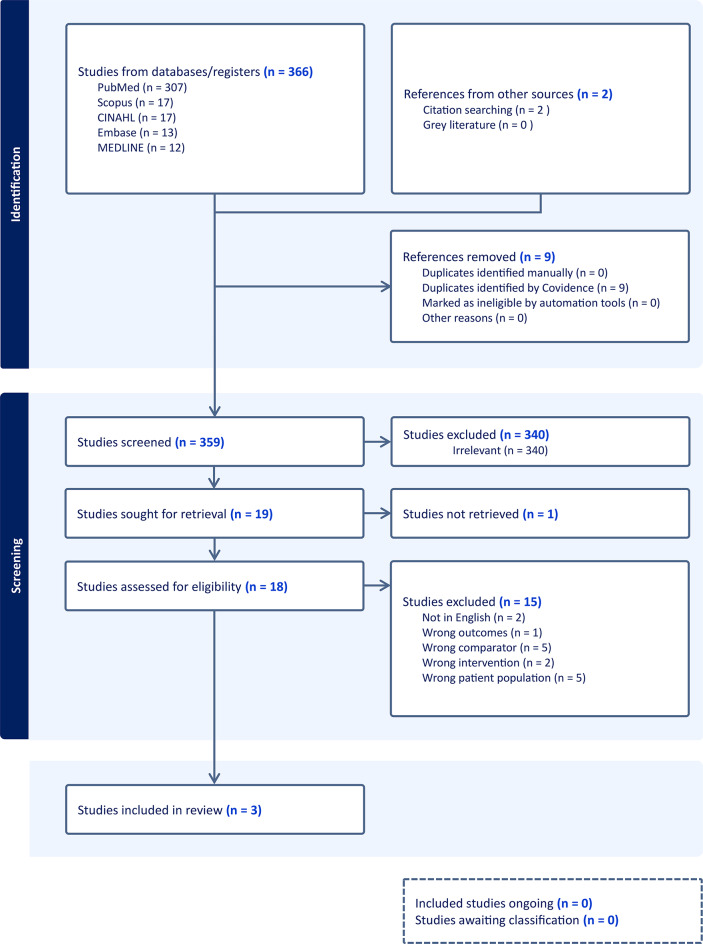
Prisma workflow

From a total of 359 studies identified during the initial search, a total of three studies, Weider et al. [43], Rothweiler et al. [44] and Duane et al. [24], were deemed relevant for inclusion in this systematic review. A majority of the studies were excluded from the initial abstract screening process as they were irrelevant to this systematic review (*n* = 340). The review process, which followed the PRISMA protocol, is detailed in Fig. 1.

### Study types

All three studies included in the present systematic review utilised a retrospective cohort design. There were no prospective or randomised controlled trials identified in the literature addressing this research question. Given the heterogeneity of included studies, meta-analysis is not appropriate.

### Sample sizes, demographic characteristics

This systematic review encompassed a total of 365 patients, with sample sizes ranging from 49 to 168. 67% of patients were male (33/49), indicating a marked gender imbalance, however, two larger studies [24, 44] did not report on gender distribution of included polytraumatised patients. The average age of the patients across two of three studies was 36.1 years old, with the final study [24] only reporting a median age of 36 across a population consisting of both operative and nonoperative groups. Only two studies reported on Injury Severity Scores (ISS) of operative patients. The current standard of practice is to report the ISS as median and interquartile range (IQR). This is due to the non-normal distribution of ISS, where the mean may not be an appropriate measure of central tendency or for comparison between different populations [45]. However, the included studies only reported ISS as averages. Rothweiler et al. [44] reported average ISS of 24.6 for the early intervention group and ISS 25.8 for delayed intervention. Similarly, Weider et al. [43] reported average ISS of 17.3 for the early intervention group and an average ISS of 18.1 for the delayed intervention group. Study specific characteristics are presented in Table 1 where available. Location of facial fractures were not analysed with respect to timing of intervention but was described. Fractures observed in the polytrauma patient population included the mandible, maxilla, zygoma, orbits, nasal bones, nasoethmoidalorbital complex and the frontal sinus. No consistent classification system was used to categorise these facial fracture patterns and/or associated soft tissue injuries.

**Table 1:** Study Characteristics

Study ID	Rothweiler	Weider	Duane
Title	Outcome and complications after treatment of facial fractures at different times in polytrauma patients	Early versus delayed repair of facial fractures in the multiply injured patient	Factors associated with delays in medical and surgical open facial fracture management.
Year	2018	1999	2022
Comparators	≤ 72 h vs. > 72 h	≤ 48 h vs. > 48 h	≤ 24 h vs. > 24 h
Country	Germany	USA	USA
Fracture Type	Mandibular, Midface, Panfacial fractures	Mandible, Maxilla, Orbital, Zygoma, Nasal area, NOE ^a^, Frontal sinus	Mandible, Nasal, Maxillary, Orbital fracture
Sample size (n)	168	49	148
Early (n)	71	7	76
Delayed (n)	97	42	72
Age (average)	30.26	28	Not reported
Average ^b^ ISS	Early 24.6, Delayed 25.8	Early 17.3, Delayed 18.1	Not reported
Follow-up duration	Not reported	Not reported	Not reported
Hosp LOS ^c^ (aver, days)	16.4 days	14.97 days	3 days
ICU LOS ^c^ (aver, days)	Not reported	5.69 days	5 days

^a^ NOE: nasoethmoidalorbital complex

^b^ ISS should be reported as median

^c^ LOS: length of stay

### Summary of studies

Weider et al. (1999) [43] described 49 polytrauma patients, with 7 patients in the early fixation (within 48 h) and 42 patients in the delayed fixation group. The average ISS was reported as 17.3 for early fixation and 18.8 for delayed fixation. However, since a true polytrauma definition generally requires an ISS of ≥ 18 combined with significant injuries from at least 2 body regions, the average ISS values near 18 suggest that the cohort may have included patients who do not strictly meet polytrauma criteria. No deaths were reported. Differences between infection rates were similar (0% early, 5% delayed) with similar ICU and hospital stay durations (5 day early, 5.8 days delayed). The authors concluded that delays related to intracranial injuries and unstable conditions did not seem to worsen outcomes, supporting a paradigm of delaying fixation to prioritise patient stability.

Rothweiler et al. (2018) [44] reported 168 polytraumatised patients with facial fractures treated operatively: 71 early (within 72 h, average ISS 24.6) and 97 delayed (average ISS 25.8). Despite slightly higher injury severity and age in the delayed group, absolute complication rates were reported to be slightly lower (21.6% vs. 25.4%), and patients in the delayed group had with fewer numbers of plate removals. Absolute infection rates were similar between groups (12.7% early, 14.4% delayed). Furthermore, mechanical (7% early, 3.1% delayed) and neurological complications (11.3% early, 9.3% delayed) were also similar. However, no statistical analysis was performed on these results to quantify statistical significance or differences between these cohorts.

Duane et al. (2022) [24] included 148 patients with open facial fractures undergoing operative management enrolled across 5 trauma centres and defined early fixation more stringently as within 24 h. The overall median ISS of the population was low, at 9 (IQR of 5–17), which likely reflects the studies large proportion of non-operative patients. However, the median ISS was not specifically reported for patients undergoing open reduction internal fixation (ORIF). They reported that severe non-facial injuries were often associated with delayed fracture intervention (OR_(vs. early ORIF)_ = 0.14, *p* < 0.01). Infection percentages were similar for both groups (6.6% early, 5.6% delayed), but no formal statistical analysis was conducted to compare between early and delayed ORIF. Similar to Weider et al. [43], delayed fixation often reflected prioritisation of other severe injuries over facial fracture outcomes.

### Time to intervention & follow-up

No studies reported on average time to operative intervention. There was a lack of standardisation in definition of what constituted as early fixation and delayed fixation, with definitions of varying from ≤ 24 h [24] (*n* = 76), to ≤ 48 h [43] (*n* = 7), and up to ≤ 72 h [44] (*n* = 71). Follow-up data was unreported across all of the included studies.

### Outcome variables & findings

Clinical outcome measures were heterogenous. Surgical site infections were the only outcome assessed by all three studies [24, 43, 44], with infection numbers ranging from 0% to 12.7% for the early intervention group and from 4.8 to 14.4% in the delayed intervention group. Less consistently reported surgical endpoints included unspecified mechanical complications (Rothweiler et al.; 7% early, 3.1% delayed [44]), unspecified ophthalmological complications (Rothweiler et al.; 5.6% early, 6.2% delayed [44]), unspecified neurological complications (Rothweiler et al.; 11.3% early, 9.3% delayed [44]), and plate removal (Rothweiler et al.; 33.8% early, 23.7% [44]). For other complications, Weider et al. [43] reported one patient each with deep vein thrombosis, chemosis and urinary tract infection in the delayed group. Healthcare-related outcomes included days on mechanical ventilation (and related pneumonia incidences), ICU length of stay (LOS), overall hospital length of stay, and mortality. These health-care related outcomes appeared comparable between the early and delayed fracture fixation groups, with average ventilator days around 3 days [44], ICU LOS around 5.2 days [24, 43] and total hospital LOS around 10.7 days [24, 43, 44, 46]. None of the studies conducted formal statistical analyses to compare outcomes between the early and delayed definitive fixation groups. These findings, including 95% confidence intervals, are detailed in Tables 2 and 3.

**Table 2:** Reported outcomes

Outcomes	Group	Study ID		
		Rothweiler	Weider	Duane
Infection (n, %)	*Early*	9/71, 12.7%	0/7, 0%	5/76, 6.6%
	*Late*	14/97, 14.4%	2/42, 4.8%	4/72, 5.6%
Mechanical complication NOS ^a^ (n)	*Early*	5/71, 7.0%	N/A	N/A
	*Late*	3/97, 3.1%	N/A	N/A
Ophthalmological complication NOS ^a^ (n)	*Early*	4/71, 5.6%	N/A	N/A
	*Late*	6/97, 6.2%	N/A	N/A
Neurological complication NOS ^a^ (n)	*Early*	8/71, 11.3%	N/A	N/A
	*Late*	9/97, 9.3%	N/A	N/A
Complication – Other ^b^ (n)	*Early*	24/71, 33.8%	0/7, 0%	N/A
	*Late*	23/97, 23.7%	3/42, 7.1%	N/A
Ventilator (days)	*Early*	N/A	3	N/A
	*Late*	N/A	3.3	N/A
VAP/Pneumonia (n)	*Early*	N/A	0/7, 0%	N/A
	*Late*	N/A	3/42, 7.1%	N/A
ICU LOS ^c^ (days)	*Early*	N/A	5	N/A
	*Late*	N/A	5.8	N/A
Hospital LOS ^c^ (days)	*Early*	N/A	16	N/A
	*Late*	N/A	14.8	N/A
Mortality (n)	*Early*	N/A	0/7, 0%	N/A
	*Late*	N/A	0/42, 0%	N/A

^a^ NOS = not otherwise specified

^b^ Other complications: Rothweiler et al. [44] – plate removal; Weider et al. [43] – deep vein thrombosis, chemosis, urinary tract infection

^c^ LOS = length of stay

**Table 3:** Reported complication rates

Outcome	Study	Early (%) [95% CI]	Late (%) [95% CI]
Surgical site infection	Rothweiler	12.7% (6.8–22.4)	14.4% (8.8–22.8)
	Weider	0.0% (0.0–35.4)	4.8% (1.3–15.8)
	Duane	6.6% (2.8–14.5)	5.6% (2.2–13.4)
Mechanical complications	Rothweiler	7.0% (3.0–15.4)	3.1% (1.1–8.7)
Ophthalmological complications	Rothweiler	5.6% (2.2–13.6)	6.2% (2.9–12.8)
Neurological complications	Rothweiler	11.3% (5.8–20.7)	9.3% (5.0–16.7)
Other complications	Rothweiler	33.8% (23.9–45.4)	23.7% (16.4–33.1)
	Weider	0.0% (0.0–35.4)	7.1% (2.5–19.0)
Ventilator-associated pneumonia	Weider	0.0% (0.0–35.4)	7.1% (2.5–19.0)
Mortality	Weider	0.0% (0.0–35.4)	0.0% (0.0–8.4)

### Risk of bias

Risk of bias assessed using the Newcastle-Ottawa Scale for Risk Bias Assessment for cohort studies [42] revealed significant risks. All cohort studies scored as “Poor” on the Newcastle-Ottawa Scale, primarily due to deficiencies in the Comparability and Outcome domains. Only Duane et al. considered the effect of confounding variables [24], by performing multivariable logistic regression to adjust for age, sex, injury mechanism, presence and severity of non-facial injuries and fracture location. However, the study still failed to meet criteria in the Outcome domain and was deemed “*Poor*” quality as per the NOS quality classification system **(**Table 4**)**.

**Table 4:** Newcastle-Ottawa Scale (NOS) Risk of Bias Assessment for Observational Studies

Study ID	Study Design	Selection (Max 4)	Comparability (Max 2)	Outcome (Max 3)	Total Score	Quality Classification
Weider, 1999	Retrospective cohort	3	0	1	**4**	Poor
Rothweiler, 2018	Retrospective cohort	3	0	1	**4**	Poor
Duane, 2022	Retrospective cohort	3	1	1	**5**	Poor

## Discussion

This systematic review evaluates the existing literature surrounding the timing of surgical fixation in maxillofacial fractures in polytraumatised patients. The review revealed that significant variations exist in the literature regarding temporal definitions of early and delayed fixation. Furthermore, outcome variables were inconsistently reported. Within the context of these limitations, early definitive fracture fixation in patients with polytrauma does not demonstrate a clear advantage over delayed fixation in surgical, healthcare-related or mortality outcomes. In the subset of studies reporting surgical site infections, rates were comparable between early intervention and delayed intervention groups in all three of four studies. Interpretation is limited by the substantial heterogeneity of studies, high risk of bias as assessed by the Newcastle-Ottawa Scale and the small subgroup sizes present (e.g. Weider et al. [43], early, *n* = 7).

### Advantages conferred by early definitive fixation

Early definitive fixation, defined as surgery within 24 h of physiological stabilisation, has demonstrated significant benefits in physiologically optimised polytrauma patients with major orthopaedic fractures [38], such as long-bone [35, 47, 48], pelvic [34], hip [49] and spinal [37, 50] fractures [38]. In stabilised patients, acute (< 24 h) open reduction internal fixation (ORIF) of pelvic and hip fractures has been associated with shorter hospital LOS, decreased red-cell transfusion requirements [51] and lower rates of respiratory complications, including incidence of adult distress respiratory syndrome (ARDS), pulmonary embolism [34, 49] and pneumonia [34, 49] compared to ORIF performed after 24 h. Early fixation of femoral fractures in stabilised patients similarly reduces the incidence of ARDS and multiorgan failure [52, 53]. Evidence also suggests a reduction in ICU LOS, hospital LOS, and decreased duration of mechanical ventilation following early fixation of long-bone fractures [35]. From a pathophysiological perspective, minimising ongoing fat and bone marrow embolisation from unstable fractures and reducing the cytokine surge (IL6, IL8, IL1β, TNF-α) [3] associated with unstabilised fractures helps to attenuate the systemic inflammatory response syndrome (SIRS) [54] and prevents the progression to multiorgan dysfunction. Early definitive fixation reduces the overall inflammatory burden, facilitating of early mobilisation, thereby reducing pulmonary complications. Although the inflammatory burden and systemic effect of unstabilised maxillofacial fractures have not been well characterised in the literature, it is plausible that these injuries trigger a smaller cytokine surge and cause a lesser degree of immune dysregulation than major orthopaedic injuries. Consequently, the decision to perform early definitive fixation in maxillofacial injuries must balance the additional surgical burden [55] against the potential systemic impact of other fractures. Contemporary trauma guidelines advocate a physiologically guided, multi-disciplinary approach to fracture fixation [3, 29]. Osseous injuries are managed in a manner which prioritises addressing those that pose a risk to life (airway compromise, uncontrolled haemorrhage, intracranial injury), then those that risk catastrophic disability or may result in significant neurological sequelae [36]. Subsequent sequential stabilisation of lower-impact fractures, including certain maxillofacial fractures, occur once the patient’s physiology has been stabilised and optimised [56]. Theoretically, a multidisciplinary approach by combining orthopaedic and maxillofacial fracture fixation in a single anaesthetic session may be considered when patient physiology, inflammatory burden and overall surgical load allow [57]. Combined early fixation of maxillofacial fractures during a single anaesthetic for other injuries may be feasible when existing facial lacerations provide for direct access to facial fractures reducing the need for further incisions and when concurrent operative fields do not require conflicting patient positions or repeated intraoperative positioning. However, in polytrauma scenarios where competing surgical priorities take precedence over lower impact maxillofacial fractures or when prolonged operative time is to be anticipated, a staged approach allowing 7–14 days for the resolution of facial oedema may be more appropriate. For complex fracture patterns, this time interval also allows for appropriate operative and logistically planning, for example, the acquisition of biomodels and/or patient specific implants, thereby improving patient outcomes. Consequently, a planned staged approach is preferable to attempting premature “early” definitive repair in these cases.

### Timing of definitive fixation in isolated maxillofacial injuries

Numerous observational studies have investigated the effects of the timing interval to definitive fracture fixation on outcomes in isolated maxillofacial trauma. Excluding cases requiring immediate surgery for life-threatening haemorrhage, airway compromise, extraocular eye muscle entrapment, or vision threatening retrobulbar haematoma, the ideal timing for surgical repair remains unclear. Several systematic reviews have highlighted the difficulties in synthesising available evidence, predominantly due to inconsistent definitions of “early” versus “delayed” fixation, ranging from 24 h to 2 weeks, and variable outcome. Limited control of confounding variables and predominance of low-level evidence hinders definitive conclusions [13, 16, 58]. Similar to that demonstrated in the current review, a few retrospective series of mandibular fractures suggest that delaying fixation does not increase surgical complications rates [59]. These studies report delay intervals of > 72 h [60], up to 5 days [61], 7 days [62] and even 13 days [63]. Facial soft tissues have a rich blood supply and contain extensive loose areolar connective tissue, which increases their susceptibility to developing marked traumatic oedema following injury [64, 65]. The presence of this oedema obscures surgical planes and bony landmarks, making precise anatomical reduction of the complex three-dimensional bony fragments challenging. These difficulties are compounded by the limited usefulness of two-dimensional intraoperative imaging for three-dimensional structures as well as the restricted availability and practicality (cost and time) of three-dimensional intraoperative imaging systems [66, 67]. Furthermore, both modalities increase patient’s exposure to ionising radiation. Therefore, in midfacial and orbital fractures, a 2-week delay is typical to allow for soft-tissue oedema to subside [25], facilitating the optimal incision placement from an aesthetic perspective and an accurate assessment of fracture reduction and alignment intra- and postoperatively [19, 68]. This is departure from early paradigms that advocated for early repair before maximal wound contracture (5–15 days), in order to allow for improved soft tissue healing over the repaired bony framework [68, 69]. Contemporary literature reflects this shift, with “delayed” fixation defined as beyond 2 weeks, and repair within this interval considered standard practice to minimise complications following orbital and midface fracture fixation [70–72]. Some authors have attempted further stratify outcomes within this 2-week timeframe. Janus et al. [73] found no significant differences in clinical outcomes when comparing between early (< 5 days) and late (> 5 days) midface fracture repair, although earlier repair trended towards increased intraoperative blood loss. This was attributed to soft tissue swelling and associated difficulty of vessel visualisation intraoperatively, resulting in inadvertent damage and subsequent haemorrhage [73]. Similarly, Jazayeri et al. [74] reported no further benefit from repairing orbital fracture repair within 24 h or 7 days compared to the conventional timeframe of within 2 weeks [74]. In complex nasoorbitoethmoid injuries, aesthetic outcomes appeared to be superior with early primary repair, which was defined as repair within 2 weeks [75]. In the current systematic review, all of the delayed cohorts underwent fixation within this two-week threshold, and it is possible that the absence of outcome disparities between the early and delayed groups likely reflects their adherence to accepted timing guidelines.

### Definitive fixation of maxillofacial fractures in polytrauma patients

Polytraumatised patients represent a unique subset of critically injured patients who present with concurrent involvement of multiple organ systems and the presence of a pronounced systemic inflammatory response [1–3]. In the polytrauma setting, surgical management of many maxillofacial injuries is often treated as a semi-elective procedure and delayed in favour of higher priority operative interventions. However, patients with polytrauma may present with primary life- or sight-threatening maxillofacial indications for surgery which cannot be delayed. These include operative intervention for fracture reduction to improve airway obstruction and aid in establishing a definitive airway; fracture reduction and/or vessel ligation to control haemorrhage refractory to conservative measures such as packing; splinting of highly unstable fractures at risk of further displacement and injury to surrounding tissue; lateral canthotomy and/or inferior septectomy to decompress the orbit in the setting of retrobulbar haemorrhage; and the repair of open globe injuries [76]. These interventions are often temporary “damage control” procedures that aim to preserve life and reduce potential disability without the risks associated with prolonged anaesthesia and surgery in a physiologically compromised patient. Once the patient has been optimised physiologically, decisions can be made about the timing of theatre for definitive management of maxillofacial trauma. Unfortunately, the studies included in the review did not comment on the distinction between early emergency management and early definitive surgical management, complicating interpretations and utility of findings regarding the effects of early treatment.

Patients with severe maxillofacial fractures also carry a high inherent risk of concurrent injuries to adjacent structures in the head and neck region, including traumatic brain injury (TBI) [7], spinal fractures, and intracranial haemorrhage [77, 78]. Reported incidence rates for these associated injuries range from 50 to 80% [79] for TBI, 12% for spinal fractures, and 17–74% [7, 79] for intracranial bleeding. In patients without immediate surgical indications, the presence of these injuries was noted to be a frequent driver of delays in definitive facial fracture fixation [24, 25, 44, 46]. Early implementation of neuroprotective measures in polytrauma patients with TBIs is essential to prevent secondary insults and development secondary brain injury [56]. Following significant intracranial trauma, the brain’s autoregulatory capacity is impaired, with a reduction of perfusion in contused regions. In concert with the brain’s increased metabolic requirements for glucose, this predisposes the brain to ischaemic injury in the initial 24 h after injury [80]. Operative management can exacerbate this vulnerability through intraoperative hypoxia, blood loss, hypotension, increase in intracranial pressure [81], all of which can further compromise cerebral perfusion [82, 83]. Numerous studies have attempted to investigate the effect of timing of definitive surgical fixation in patients with TBIs but have found conflicting evidence [28, 77, 82, 83]. Current guidelines recommend early definitive treatment of major fractures in cases of mild TBI and to delay definitive fracture fixation until after appropriate neurosurgical interventions in cases where significant intracranial pathology is present [27, 36]. Clinically, intracranial pressure monitoring serves as a surrogate measure of cerebral perfusion pressures [84]. These considerations have also been reported in maxillofacial literature, with Duane et al. [24] reporting that concomitant serious head injury (OR 0.14, *p* < 0.01) and moderate/severe TBI (OR = 0.10, *p* < 0.01) were both associated with delayed definitive maxillofacial fracture fixation amongst a cohort of polytrauma patients. Apart from the immediate surgical indications discussed previously, the management of life-threatening brain injuries often takes priority, especially if certain maxillofacial fractures can tolerate delays of up to two weeks (as discussed previously). Therefore, it is unsurprising that TBIs are one of the principal determinants of timing for definitive fracture repair in many circumstances of polytraumatised patients.

### Limitations

This systematic review has several important limitations. All studies employed a retrospective design, which introduced inherent selection and information biases, therefore limiting the ability to establish causal relationships. The absence of standardised inclusion criteria across studies created substantial heterogeneity in both the definition and characterisation of the polytrauma population. The ISS values were reported as averages (not methodologically appropriate) and the borderline values, pose the question of whether these populations are representative of “true” polytrauma. Similarly, heterogeneity was present in the classification of maxillofacial fractures, including whether these fractures were open or closed. Furthermore, the definition of “early” versus “delayed” surgical intervention varied considerably across studies, compounding temporal comparisons. The included studies also did not stratify patients according to surgical indication, specifically whether early fixation was undertaken for emergency indications (such as airway compromise from displaced mandibular fractures, uncontrolled haemorrhage, severely displaced fractures with extensive soft tissue injury, or vision-threatening retrobulbar haematomas) versus elective timing in physiologically stable patients. This limits the accurate interpretation of observed outcomes in the early group, as the true benefits or harms of early definitive fixation are likely confounded by the higher risks associated with emergency intervention in unstable patients. Outcome reporting was heterogenous, with limited standardisation of clinically relevant endpoints. This was quite evident in the lack of granularity or nuance of reporting of maxillofacial specific clinical, surgical and functional outcomes. No studies reported on malocclusion, trigeminal nerve dysfunction, mouth opening, diplopia, degree of mouth opening, masticatory function, occlusal relationship or anatomical reduction of maxillofacial fractures in the polytrauma population. Outcomes and complications were discussed in broad categories, such as infection, or mechanical, neurological and ophthalmological complications without specifying what these complications were. This makes it difficult to interpret these findings and apply them specifically to a maxillofacial context. Clinical outcomes including multiple organ failure (MOF), sepsis, TBI progression, ventilator days, ICU LOS and hospital LOS were inconsistently reported. Confounders were poorly accounted for, with only Duane et al. [24] performing multivariable adjustments. Furthermore, the evolving concept and definition of “polytrauma” over the past 15 years [2, 85] complicates comparisons in existing literature, older studies may have employed different diagnostic criteria or injury severity thresholds, rendering patient populations incomparable to each other and weakening external validity.

Despite the preliminary reassuring finding of comparable rates of infection and other complications between early and delayed maxillofacial fixation group, definitive conclusions about optimal and safe timing requires confirmation through robust prospective, randomised, multicentre trials that use standardised of polytrauma, fracture classifications, surgical timing and outcome measures. Future studies aiming to investigate the optimal timing of definitive facial fracture fixation in the polytrauma population should include the following components: (1) a prospective study design with standardised inclusion criteria for polytrauma (i.e. Berlin Definition [2]); (2) uniformly defined time intervals for early versus delayed fixation (i.e. ≤48 h vs. > 48 h); (3) standardised outcome measures for clinical and hospital related outcomes, including specific maxillofacial complications; (4) defined and adequate follow-up periods and lastly, (5) appropriate multivariable adjustment for confounders.

## Conclusion

The current limited evidence on timing of definitive surgical stabilisation of maxillofacial fractures is based on low-quality retrospective studies without clear and uniform definition of patient population (polytrauma and facial fracture severity), consensus of the definition of early timing and standardised outcome measures. There is no evidence to support the superiority of the currently practiced delayed approach to definitive maxillofacial fracture management. As a substantial gap in knowledge exists, especially in the context of modern traumatic shock resuscitation strategies, high-quality prospective observational and potentially randomised trials addressing the current design limitations in inclusions, definition and outcomes are required to establish the optimal timing for surgery in polytrauma.

## Supplementary Information

Below is the link to the electronic supplementary material.

Supplementary Material 1

## Data Availability

No datasets were generated or analysed during the current study.
