# Supplementary material for: Early versus delayed fixation of maxillofacial fractures in polytrauma: a systematic review
Source: Eur J Trauma Emerg Surg. 2026 Feb 23;52(1):58. doi: 10.1007/s00068-026-03127-2 (PMC12929254; doi:10.1007/s00068-026-03127-2)
Supplement: Supplementary file 1 — Supplementary Material 1 [file 68_2026_3127_MOESM1_ESM.docx]

**Supplementary Table 1: Ovid MEDLINE(R) All, 1946 to July 20, 2025**

| **Search no.** | **Search Terms** | **Results (n)** |
| --- | --- | --- |
| 1 | Facial Bones.mp. or exp Facial Bones/ | 146497 |
| 2 | Maxillofacial Injuries.mp. or Maxillofacial Injuries/ | 4789 |
| 3 | Zygomatic Fractures.mp. or exp Zygomatic Fractures/ | 2023 |
| 4 | Mandibular Fractures.mp. or Mandibular Fractures/ | 8153 |
| 5 | Orbital Fractures.mp. or Orbital Fractures/ | 4474 |
| 6 | Nasal Bone/ or Nasal Bone fractures.mp. | 3241 |
| 7 | Le Fort Fracture.mp. or Maxillary Fractures/ | 1726 |
| 8 | (midfacial fracture* or midface fracture* or mandibular fracture* or jaw fracture* or zygomatic fracture* or orbital fracture* or NOE fracture* or naso-orbito-ethmoidal fracture* or nasal fracture* or maxillary fracture* or Le Fort fracture* or facial fracture*).mp. [mp=title, book title, abstract, original title, name of substance word, subject heading word, floating sub-heading word, keyword heading word, organism supplementary concept word, protocol supplementary concept word, rare disease supplementary concept word, unique identifier, synonyms, population supplementary concept word, anatomy supplementary concept word] | 18550 |
| 9 | Facial Injuries/ or facial fractures.mp. | 8788 |
| 10 | surgical procedure.mp. or Surgical Procedures, Operative/ | 113828 |
| 11 | Fracture Fixation.mp. or Fracture Fixation/ | 76073 |
| 12 | (Surgical Repair or fixation).mp. [mp=title, book title, abstract, original title, name of substance word, subject heading word, floating sub-heading word, keyword heading word, organism supplementary concept word, protocol supplementary concept word, rare disease supplementary concept word, unique identifier, synonyms, population supplementary concept word, anatomy supplementary concept word] | 293079 |
| 13 | Open Fracture Reduction/ or Fracture Fixation, Internal/ or ORIF.mp. | 47215 |
| 14 | Time Factors/ or timing.mp. | 1417669 |
| 15 | early repair.mp. | 1046 |
| 16 | delayed repair.mp. | 920 |
| 17 | early surgery.mp. or Time-to-Treatment/ | 15568 |
| 18 | delayed surgery.mp. | 1972 |
| 19 | surgical timing.mp. | 1388 |
| 20 | timing of repair.mp. | 308 |
| 21 | treatment delay.mp. or Treatment Delay/ | 2752 |
| 22 | (outcome* or function* or occlusion or "mouth opening" or diplopia or "nasal patency" or "aesthetic outcome" or enophthalmos or symmetry or complication* or infection or malunion or reoperation or satisfaction or cost).mp. [mp=title, book title, abstract, original title, name of substance word, subject heading word, floating sub-heading word, keyword heading word, organism supplementary concept word, protocol supplementary concept word, rare disease supplementary concept word, unique identifier, synonyms, population supplementary concept word, anatomy supplementary concept word] | 12647395 |
| 23 | Treatment Outcome/ or outcome.mp. | 2570556 |
| 24 | function.mp. | 2874191 |
| 25 | occlusion.mp. or Dental Occlusion/ | 217239 |
| 26 | anatomical reduction.mp. | 1631 |
| 27 | Postoperative Complications/ or complication.mp. | 768386 |
| 28 | reoperation.mp. or Reoperation/ | 126293 |
| 29 | Fractures, Malunited/ or malunion.mp. | 5335 |
| 30 | 14 or 15 or 16 or 17 or 18 or 19 or 20 or 21 | 1431981 |
| 31 | 1 or 2 or 3 or 4 or 5 or 6 or 7 or 8 or 9 | 165500 |
| 32 | 10 or 11 or 12 or 13 | 403723 |
| 33 | 14 or 15 or 16 or 17 or 18 or 19 or 20 or 21 | 1431981 |
| 34 | 22 or 23 or 24 or 25 or 26 or 27 or 28 or 29 | 12647864 |
| 35 | 31 and 32 and 33 and 34 | 491 |
| 36 | exp Multiple Trauma/ or multi trauma.mp. or polytrauma.mp. | 17522 |
| 37 | Respiration, Artificial/ or Ventilators, Mechanical/ or ventilat*.mp. or Ventilation/ | 262881 |
| 38 | 36 or 37 | 279432 |
| 39 | 35 and 38 | 13 |

**Supplementary Table 2: EMBASE - 1947 to July 20, 2025**

| **Search no.** | **Search Term** | **Results (n)** |
| --- | --- | --- |
| 1 | facial bone fracture.mp. or face fracture/ | 3842 |
| 2 | midface fracture.mp. or exp midface fracture/ or maxillofacial injury/ or exp mandible fracture/ or face injury/ or exp zygoma arch fracture/ | 27811 |
| 3 | zygomatic fracture.mp. or exp zygomatic fracture/ or exp orbital fracture/ or orbital fracture.mp. or exp nasal bone fracture/ or nasal bone fracture.mp. or exp le fort fracture/ or le fort fracture.mp. | 10169 |
| 4 | (midface fracture* or midfacial fracture* or mandibular fracture* or jaw fracture* or NOE fracture* or naso-orbito-ethmoidal fracture*).mp. [mp=title, abstract, heading word, drug trade name, original title, device manufacturer, drug manufacturer, device trade name, keyword heading word, floating subheading word, candidate term word] | 7196 |
| 5 | exp surgical procedure/ or surgical procedure.mp. or exp fracture fixation/ or fracture fixation.mp. or exp open reduction internal fixation/ or open reduction internal fixation.mp. or exp ORIF/ or ORIF.mp. or exp surgical repair/ or surgical repair.mp. or exp internal fixation/ or internal fixation.mp. [mp=title, abstract, heading word, drug trade name, original title, device manufacturer, drug manufacturer, device trade name, keyword heading word, floating subheading word, candidate term word] | 7287899 |
| 6 | early repair.mp. or exp early repair/ or delayed repair.mp. or exp delayed repair/ or surgical timing.mp. or exp surgical timing/ or treatment delay.mp. or exp treatment delay/ or early surgery.mp. or exp early surgery/ or delayed surgery.mp. or exp delayed surgery/ | 36407 |
| 7 | Outcome.mp. or exp outcome/ or aesthetic outcome.mp. or exp aesthetic outcome/ or function*.mp. or exp function/ or dental occlusion.mp. or exp dental occlusion/ or diplopia.mp. or mouth opening.mp. or exp mouth opening/ or enophthalmos.mp. or exp enopthalmos/ or infection.mp. or exp infection/ or malunion.mp. or exp malunion/ or reoperation.mp. or exp reoperation/ or nerve damage.mp. or exp nerve damage/ or cost.mp. or satisfaction.mp. or exp satisfaction/ | 16652112 |
| 8 | 1 or 2 or 3 or 4 | 37862 |
| 9 | 5 and 6 and 7 and 8 | 159 |
| 10 | exp Multiple Trauma/ or multi trauma.mp. or polytrauma.mp | 22008 |
| 11 | exp ventilator/ or ventilat*.mp. or exp artificial ventilation/ | 568191 |
| 12 | ICU.mp. or exp intensive care unit/ or intensive care/ | 571554 |
| 13 | 10 or 11 or 12 | 1015591 |
| 14 | 8 and 9 and 13 | 13 |

**Supplementary Table 3: Scopus, to July 20, 2025**

| **Search Terms** | **Results (n)** |
| --- | --- |
| “maxillofacial injuries” OR "midface fracture" OR "midfacial fracture" OR "mandibular fracture" OR "jaw fracture" OR "zygomatic fracture" OR “zygomaticomaxillary complex fracture” OR "orbital fracture" OR "nasal fracture" OR "maxillary fracture" OR "Le Fort fracture" OR "NOE fracture" OR “nasoethmoid fracture” |  |
| **AND** | |
| "early repair" OR "delayed repair" OR "early surgery" OR "delayed surgery" OR "surgical timing" OR "treatment delay" |  |
| **AND** | |
| "surgical repair" OR "ORIF" OR "open reduction" OR "internal fixation" OR “operative” OR “operation” OR “surgical procedure” |  |
| **AND** | |
| outcome OR functional outcome OR aesthetic outcome OR dental occlusion OR diplopia OR mouth opening OR enophthalmos OR complication OR infection OR reoperation OR malunion OR satisfaction OR cost | 229 |
| **AND** | |
| Polytrauma OR Multi-trauma OR multi trauma OR multi injur* OR multitrauma OR multi injur* OR ICU OR intensive care* OR ventilat* | 17 |

**Supplementary Table 4: CINAHL, 1937 to July 20, 2025**

| **Search Terms** | **Results (n)** |
| --- | --- |
| (MH "Facial Injuries+") OR (MH "Mandibular Fractures") OR (MH "Maxillofacial Injuries") OR (MH "Orbital Fractures") |  |
| **OR** |  |
| maxillofacial fracture OR facial fracture OR nasal fracture OR nasoethmoid fracture OR maxillary fracture OR maxilla fracture OR le fort fracture OR mandible fracture OR mandibular fracture OR orbit fracture OR orbital fracture OR midface fracture OR midfacial fracture OR NOE fracture or zygomatic fracture OR zygomaticomaxillary complex fracture |  |
| **AND** | |
| (MH "Fracture Fixation") OR (MH "Surgery, Operative") OR ORIF OR "internal fixation" OR "surgical repair" OR “open reduction” OR fracture repair OR operation OR operative |  |
| **AND** | |
| ("early surgery" OR "delayed surgery" OR "timing of repair" OR "treatment delay") |  |
| **AND** | |
| (functional outcome OR aesthetic outcome OR dental occlusion OR diplopia OR mouth opening OR enophthalmos OR infection OR reoperation OR cost OR satisfaction) | 41 |
| **AND** | |
| ((polytrauma*) OR (multi-trauma*) OR (MH "multi trauma) or (MH "poly trauma) OR (MH "multi injury") OR ("ventilat*") OR (multi trauma*) OR (multi injur*) OR (MH "ICU") OR (MH "intensive care*")) | 17 |

**Supplementary Table 5: PubMed, to July 20, 2025**

| **Search Terms** | **Results (n)** |
| --- | --- |
| "Facial Bones"[Mesh] OR "Maxillofacial Injuries"[Mesh] OR "Fractures, Bone"[Mesh] OR "Zygomatic Fractures"[Mesh] OR "Mandibular Fractures"[Mesh] OR "Orbital Fractures"[Mesh] OR "Nasal Bone"[Mesh] OR "Le Fort Fractures"[Mesh] |  |
| **OR** |  |
| midfacial fracture* OR midface fracture* OR mandibular fracture* OR jaw fracture* OR zygomatic fracture* OR zygomaticomaxillary complex fracture* OR orbital fracture* OR NOE fracture* OR naso-orbito-ethmoidal fracture* OR nasal fracture* OR maxillary fracture* OR Le Fort fracture* OR facial fracture* |  |
| **AND** | |
| "Surgical Procedures, Operative"[Mesh] OR "Fracture Fixation"[Mesh] OR "Surgical Repair"[tiab] OR fixation[tiab] OR ORIF[tiab] OR "open reduction"[tiab] OR "internal fixation"[tiab] |  |
| **AND** | |
| timing[tiab] OR "early repair"[tiab] OR "delayed repair"[tiab] OR "early surgery"[tiab] OR "delayed surgery"[tiab] OR "surgical timing"[tiab] OR "timing of repair"[tiab] OR "treatment delay"[tiab] |  |
| **AND** | |
| timing[tiab] OR "early repair"[tiab] OR "delayed repair"[tiab] OR "early surgery"[tiab] OR "delayed surgery"[tiab] OR "surgical timing"[tiab] OR "timing of repair"[tiab] OR "treatment delay"[tiab] |  |
| **AND** | |
| outcome*[tiab] OR function*[tiab] OR occlusion[tiab] OR "mouth opening"[tiab] OR diplopia[tiab] OR "nasal patency"[tiab] OR "aesthetic outcome"[tiab] OR enophthalmos[tiab] OR symmetry[tiab] OR complication*[tiab] OR infection[tiab] OR malunion[tiab] OR reoperation[tiab] OR satisfaction[tiab] OR cost[tiab] | 1650 |
| **AND** | |
| Multitrauma OR Polytrauma OR multiple injuries* OR ICU OR intensive care OR ventilat* OR multi-injur* OR Multi*trauma | 306 |
